# Influence of Sub-Surface Irrigation on Soil Conditions and Water Irrigation Efficiency in a Cherry Orchard in a Hilly Semi-Arid Area of Northern China

**DOI:** 10.1371/journal.pone.0073570

**Published:** 2013-09-09

**Authors:** Gao Peng, Wang Bing, Zhang Guangcan

**Affiliations:** 1 Shandong Agricultural University, College of Forestry/Taishan Mountain Forest Ecosystem Research Station/Shandong Provincial Key Laboratory of Soil Erosion and Ecological Restoration, Tai’an, Shandong, P. R. China; 2 Research Institute of Forest Ecology Environmental Protection, Chinese Academy of Forestry, Beijing, P. R. China; DOE Pacific Northwest National Laboratory, United States of America

## Abstract

Sub-surface irrigation (SUI) is a new water-saving irrigation technology. To explore the influence of SUI on soil conditions in a cherry orchard and its water-saving efficiency, experiments were conducted from 2009 to 2010 using both SUI and flood irrigation (FLI) and different SUI quotas in hilly semi-arid area of northern China. The results demonstrated the following: 1) The bulk density of the soil under SUI was 6.8% lower than that of soil under FLI (P<0.01). The total soil porosity, capillary porosity and non-capillary porosity of soils using SUI were 11.7% (P<0.01), 8.7% (P<0.01) and 43.8% (P<0.01) higher than for soils using FLI. 2) The average soil temperatures at 0, 5, 10, 15 and 20 cm of soil depth using SUI were 1.7, 1.1, 0.7, 0.4 and 0.3°C higher than those for FLI, specifically, the differences between the surface soil layers were more significant. 3) Compared with FLI, the average water-saving efficiency of SUI was 55.6%, and SUI increased the irrigation productivity by 7.9-12.3 kg m^-3^ ha^-1^. 4) The soil moisture of different soil layers using SUI increased with increases in the irrigation quotas, and the soil moisture contents under SUI were significantly higher in the 0-20 cm layer and in the 21-50 cm layer than those under FLI (*P*<0.01). 5) The average yields of cherries under SUI with irrigation quotas of 80-320 m^3^ ha^-1^ were 8.7%-34.9% higher than those in soil with no irrigation (CK2). The average yields of cherries from soils using SUI were 4.5%-12.2% higher than using FLI. It is appropriate to irrigate 2-3 times with 230 m^3^ ha^-1^ per application using SUI in a year with normal rainfall. Our findings indicated that SUI could maintain the physical properties, greatly improve irrigation water use efficiency, and significantly increase fruit yields in hilly semi-arid areas of northern China.

## Introduction

The global water crisis is one of the major environmental concerns in the world. Especially in hilly semi-arid and arid regions, water resources are the most strongly limiting factors for the growth of plants [1–3]. To cope with water shortages, it is necessary to adopt water-saving agricultural countermeasures. The efficient use of irrigation water is becoming increasingly important. Many countries have increasingly made scientific efforts to solve this problem [4–7].

Agricultural water conservation programs will dominate these efforts because agriculture uses most of the available water, including over 80% in China [8–10]. Some conservation strategies that can help improve future water availability include optimum irrigation scheduling techniques employing monitoring of soil moisture and weather, the use of drought-tolerant crop varieties, mulches, the leveling of land to ensure uniform water delivery, improving water delivery infrastructure by lining canals and/or replacing canals with pipe systems, and the use of sub-surface irrigation (SUI) methods [11].

SUI systems are capable of applying small amounts of water directly to the plant root zone where the water is needed, and these small amounts can be applied frequently to maintain favorable moisture conditions in the root zone. Some of the potential benefits of SUI are improvements in yield and quality and the reduction of production costs [12,13].

SUI is used primarily in the United States, Australia, and Israel, generally for irrigating greenhouses, field crops and urban greenspaces. In China, SUI is used mainly for irrigating high-value vegetable crops, such as onions (*Allium cepa*), cantaloupes (*Cucumis melo*) and watermelon ( *Citrulus* *lanatus* ). In recent years, fruit tree production has become an important way for farmers to increase their income in the hilly semi-arid and arid areas of China. However, water shortages remain an important limiting factor for fruit tree production, so SUI is used in many orchards in these hilly semi-arid and arid areas. At present, most research on SUI theory and technology has focused on water movement and adjustment and on the control parameters of irrigation, as well as on water-saving effects in solar greenhouses, in recent years [14–16]. Some scholars have studied the effects of SUI on wheat or cotton in the field [17–19]. However, systematic studies on the influence of SUI on soil conditions in orchards in hilly semi-arid and arid areas of China and the water-saving irrigation efficiency of SUI have been rare [20].

The purpose of the present study was to explore the influence of SUI on soil conditions and its water-saving irrigation efficiency in a cherry orchard in a hilly semi-arid and arid region. Experiments were conducted from 2009 to 2010 under both SUI and flood irrigation (FLI) and using different SUI quotas in a hilly semi-arid area of Shandong province, which might not only provide a scientific basis for the efficient use of water resources but also for adopting rational water-saving irrigation measures in orchards.

## Materials and Methods

### Ethics Statement

The research station for this study is owned by Shandong Agricultural University. This study was approved by Taishan Mountain Forest Ecosystem Research Station and the Shandong Provincial Key Laboratory of Soil Erosion and Ecological Restoration.

### Study Area

The experiment was conducted from 2009 to 2010 using both SUI and FLI in a cherry orchard located in a hilly semi-arid area of Shandong Province in northern China (116° 43′-117°44′ E, 36° 14′-37°15′ N, 205 m altitude), which has a warm, temperate, continental, semi-humid monsoon climate. The soil type is brown soil, and the soil pH is between 6.0 and 7.5. The soil texture is mainly sandy loam with a high proportion of sand. The soil moisture properties of the different soil layers are presented in Table 1.

**Table 1:** Soil basic properties of the different layers at the study area.

Soil depth(cm)	Soil texture ^†^			Soil hydraulic conductivity (mm·h^-1^)	Permanent wilting point (%)	Soil water capacity (%)	Capillary capacity (%)
	Sand (%)	Silt (%)	Clay (%)				
0-20	71.2	10.2	18.6	17.2	7.67	21.86	35.12
21-50	67.3	12.0	20.7	16.6	8.24	19.65	28.32
51-80	64.1	13.8	22.1	16.1	8.98	20.16	30.21
81-110	61.3	14.4	24.3	15.4	9.63	20.62	29.89

“†” According to the soil texture classification criteria of international system, the numbers are the proportion of Sand (2-0.02mm), Silt (0.02-0.002mm) and Clay (<0.002mm).

### Materials and layout of SUI pipes

**Figure 1:**
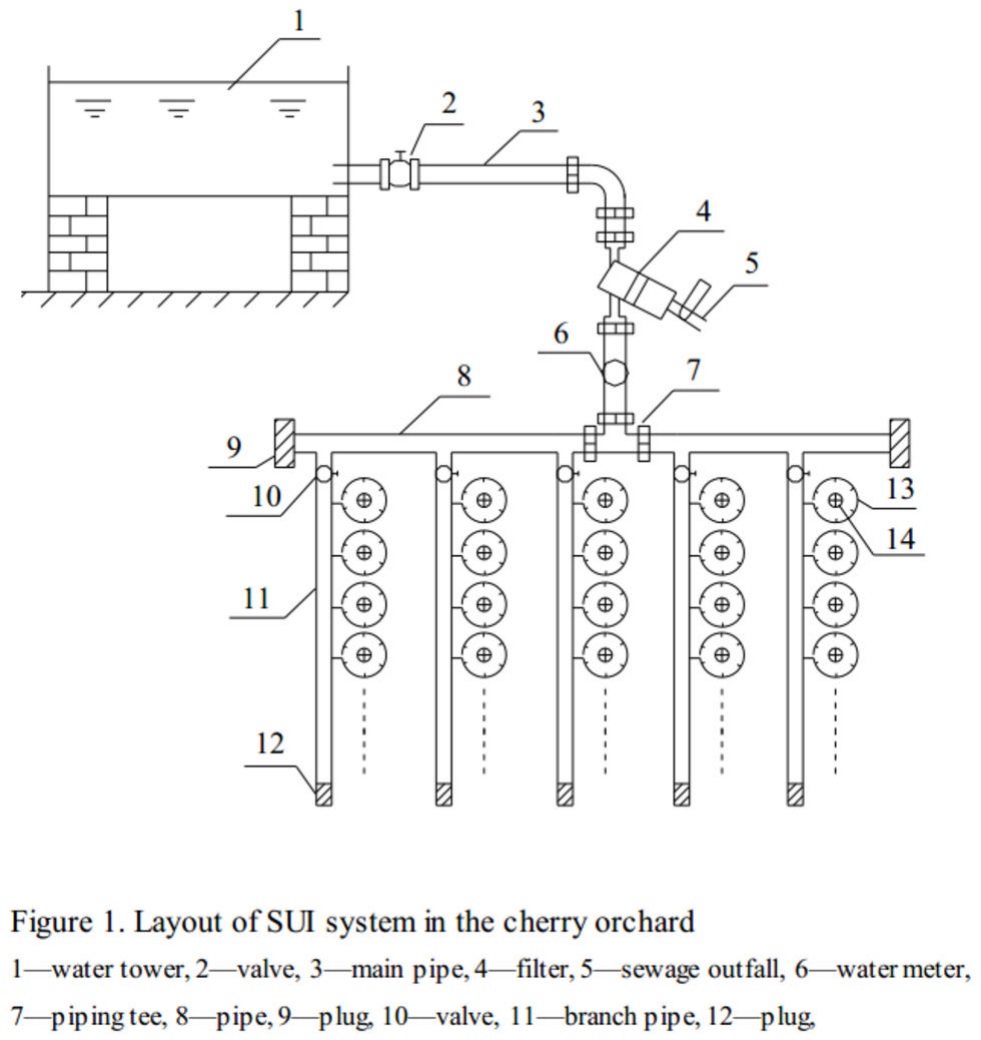
Layout of SUI system in the cherry orchard.

The cherry trees were 5 years old and had been planted in north–south rows, with a 3.0 m within–row spacing and 4 m between the rows. The SUI pipes (external diameter was 21 mm and internal diameter was 20 mm) were buried at a depth of 30 cm, surrounding the trunk and in a circle, the diameter of which approximately equaled the crown width. The layout of the SUI system in the cherry orchard is presented in Figure 1.

### Two irrigation methods and effects of water supplementation

In this study, two irrigation methods (SUI and FLI) were selected for irrigating the cherry trees, and FLI was designated as the control (CK1). The experimental plots were designed with a randomized block design. Each plot was 12 m in length, 8 m in width and had a total area of 96 m^2^.

The total annual rainfall in 2009 (a year of normal rainfall) and 2010 (a year of low rainfall) was 610.5 mm and 515.6 mm, respectively, while during the growth period, the rainfall was 510.2 mm and 432.5 mm, respectively. The cherry trees were irrigated on April 26th and May 18th in 2009 (two irrigations) and on April 15th, May 20th and June 5th in 2010 (three irrigations). The amounts of water used for each irrigation using SUI and FLI were 320 m^3^ ha^-1^ and 720 m^3^ ha^-1^, respectively.

### Different amounts of irrigation water for cherry trees using SUI or FLI

Five different irrigation water levels (80 m^3^ ha^-1^, 150 m^3^ ha^-1^, 230 m^3^ ha^-1^, 320 m^3^ ha^-1^ and 0 m^3^ ha^-1^ (no irrigation, CK2)) were examined for both SUI and FLI. Each experimental plot was 12 m long and 8 m wide, with a total area of 96 m^2^. The amount of irrigation water was determined by the water requirements of cherry trees for normal growth in the research area and the total annual rainfall situation in 2009 and 2010 in the research area, especially the rainfall during the growth period. The cherry trees were irrigated by both SUI and FLI methods on April 26th and May 18th in 2009 and on April 15th, May 20th and June 5th in 2010.

### Experimental observations and statistical analysis

After the irrigation experiment had been completed, soil moisture was monitored by a Time-Domain Reflector (TDR, America). The soil around the cherry tree roots was sampled using the ring sampling method, and each plot was measured three times. The soil bulk density of the plots was determined using ring sampling and by measuring the dry weight of the soil [21], separately, in unit volumes, at depths of 0-20, 21-50, 51-80 and 81-110 cm. The total soil porosity can be divided into the soil capillary porosity and non-capillary porosity, that is, the total soil porosity is equal to the sum of the soil capillary porosity and non-capillary porosity. Using the ring knife series analysis method [22,23], determination of the total soil porosity and soil capillary porosity, and then the non-capillary porosity is equal to the diffence between the total soil porosity and soil capillary porosity. Soil temperatures at 0, 5, 10, 15 and 20 cm of soil depth were measured using a soil geothermometer (11060, America). The amount of irrigation was determined by a water meter (DN15-50, Nanjing, Jiangsu, China). The baseline values of the physical characteristics of the soil were measured before the first irrigation, and the growth of the trees and the cherry yield were measured after irrigation.

Significances were tested by a one-way ANOVA, followed by Duncan’s test at P<0.05 and 0.01 by using SPSS-PC statistical software [24], and the results are expressed as the mean values ± SE of three observations for each plot.

## Results

### Changes in soil bulk density and soil porosity

The physical properties of the soil were different between the SUI and FLI treatments (CK1) (Table 2). The bulk density of the soil from different layers of the SUI plots was lower from those using FLI, and the average value was 6.8% lower (P<0.01). The total soil porosity, capillary porosity and non-capillary porosity of the different layers from the SUI plots were higher than from the FLI plots; the average values were 11.7%, 8.7% and 43.8% higher, respectively. Moreover, Table 2 showed that the soil bulk density at 0-20cm soil layers from SUI was 9.1% lower than that from FLI and the total soil porosity, capillary porosity and non-capillary porosity were 15.8%, 12.6% and 49.3% higher(*P*<0.01) than that from FLI. The soil bulk density at 21-50cm soil layers from SUI was 7.5% lower than that from FLI and the total soil porosity, capillary porosity and non-capillary porosity were 8.6%, 7.9% and 21.7% higher(*P*<0.01) than that from FLI. The soil bulk density at 51-80cm soil layers from SUI was 6.7% lower than that from FLI and the total soil porosity, capillary porosity and non-capillary porosity were 11.0%, 7.0% and 51.8% higher(*P*<0.01) than that from FLI. The soil bulk density at 81-110cm soil layers from SUI was 3.3% lower than that from FLI and the total soil porosity, capillary porosity and non-capillary porosity were 11.2%, 7.4% and 48.0% higher(*P*<0.01) than that from FLI. In addition, hardening and cracking were also found on the soil surface under FLI, whereas they were not found under SUI. The average width of the cracks in the soil was 4.12 mm (Table 2), while the maximum was 5.23 mm and appeared 3 days after FLI. These observations indicated that SUI had a greater ability to maintain soil structure than did FLI.

**Table 2:** Physical properties of soil under SUI and FLI (CK1)^†^.

Irrigation method	Depth of soil layer(cm)	Soil bulk density (g·cm^-3^)	Total soil porosity (%)	Capillary porosity (%)	Non-capillary porosity (%)	Average width of soil cracking (mm)
SUI	0-20	1.30±0.07^a^	46.76±3.35^a^	41.43±2.89^a^	5.33±0.68^a^	0.00
	21-50	1.35±0.08^a^	44.95±3.18^b^	40.35±2.41^b^	4.83±0.54^a^	
	51-80	1.40±0.10^b^	44.48±3.11^b^	39.65±2.26^bc^	4.60±0.47^ab^	
	81-110	1.47±0.13^c^	43.56±3.09^b^	39.15±2.18^c^	4.41±0.41^b^	
	Average value	1.38±0.10^a^	44.94±3.18^ab^	40.15±2.44^b^	4.79±0.53^a^	
FLI(CK1)	0-20	1.43±0.11^b^	40.38±2.45^cd^	36.81±2.10^b^	3.57±0.31^d^	4.12
	21-50	1.46±0.13^bc^	41.38±2.87^c^	37.41±2.08^d^	3.97±0.38^c^	
	51-80	1.50±0.14^cd^	40.08±2.63^de^	37.05±2.13^d^	3.03±0.27^e^	
	81-110	1.52±0.15^d^	39.19±2.12^e^	36.46±2.02^e^	2.98±0.23^e^	
	Average value	1.48±0.13^c^	40.25±2.52^d^	36.93±2.08^de^	3.33±0.30^de^	

Different small letter means *P*<0.01. “†” The numbers in Table 2 are the average values under SUI and FLI in 2009 and 2010. SUI is the abbreviation of subsurface irrigation, it is water-saving irrigation way of capable of applying small amounts of water directly to the plant root zone where the water is needed. FLI is the abbreviation of flood irrigation, it is a wild flooding irrigation way. FLI is selected as control (CK1), it is in order to compare with SUI. The baseline values of test soil physical characteristics before the first irrigation: soil bulk density 1.39 g·cm^- 3^; total soil porosity 43.62%; capillary porosity 39.18%; non-capillary porosity 4.11%.

### Changes in soil temperature

**Figure 2:**
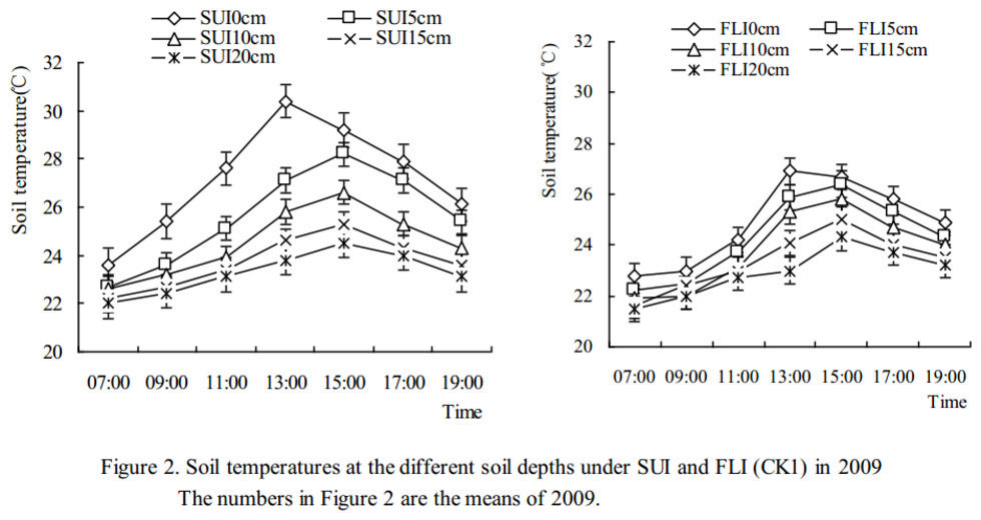
Soil temperatures at the different soil depths under SUI and FLI (CK1) in 2009.

**Figure 3:**
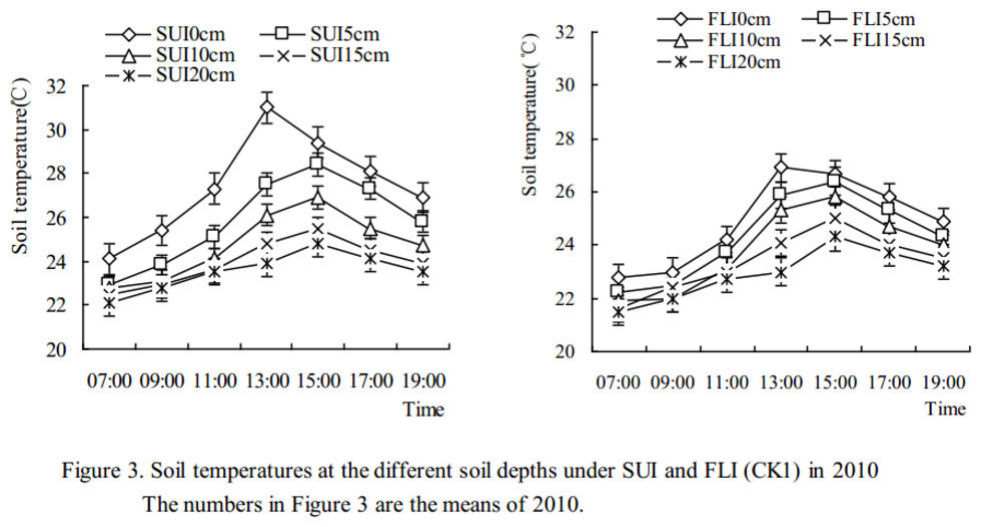
Soil temperatures at the different soil depths under SUI and FLI (CK1) in 2010.

Soil temperatures in the 0-20 cm soil layer were measured every two hours from 7: 00 to 19: 00 for 3 days after soils were irrigated on April 26th and May 18th in 2009 and April 15th, May 20th and June 5th in 2010. Changes in soil temperature at the different soil depths for SUI and FLI are presented in Figure 2 (2009) and Figure 3 (2010). Figure 2 indicates that soil temperatures in the different soil depth layers after SUI were higher than those from FLI, that the average differences were 1.6, 0.9, 0.6, 0.4, and 0.2°C at 0, 5, 10, 15, and 20 cm of soil depth, respectively, and that the maximum value was 3.4°C and occurred on the soil surface at 13: 00. Figure 3 indicates that the average soil temperature differences were 1.7, 1.1, 0.7, 0.4, and 0.3°C at 0, 5, 10, 15, and 20 cm of soil depth, respectively, and that the maximum value was 4.1°C and occurred on the soil surface at 13: 00. The ANOVA indicated that the differences in soil temperatures at 0 cm and 5 cm of soil depth for SUI and FLI were extremely significant (for 0 cm *P*<0.01; for 5 cm *P*<0.01) and that a significant difference existed at the 10 cm soil depth (*P*<0.05), whereas no significant differences were observed at the 15 cm and 20 cm soil depths (for 15 cm *P*>0.05; for 20 cm *P*>0.05).

### Water-saving effects of SUI and its irrigation productivity

Irrigation quotas and irrigation productivity under both SUI and FLI are presented in Table 3. The total amount of irrigation water used in SUI and FLI was 640.0 m^3^ ha^-1^ and 1,440.0 m^3^ ha^-1^ in 2009, respectively, and the irrigation productivities were 21.96 kg m^-3^ ha^-1^ and 9.66 kg m^-3^ ha^-1^, respectively. In 2010, the irrigation quotas for SUI and FLI were 960.0 m^3^ ha^-1^ and 2,160.0 m^3^ ha^-1^, respectively, while the irrigation productivities were 13.7 kg m^-3^ ha^-1^ and 5.78 kg m^3^ ha^-1^, respectively. When compared with FLI, SUI could save 800-1,200 m^3^ ha^-1^ of water. Its average water-saving efficiency was 55.6%. The irrigation productivity of SUI was 7.92-12.30 kg m^3^ ha^-1^ higher than was that of FLI. The ANOVA indicated that the differences in water-saving efficiency and irrigation productivity between SUI and FLI were all extremely significant (*P*<0.01). This analysis demonstrated that SUI had a higher water-saving efficiency than did FLI and that SUI improved the efficiency of irrigation water use to some extent.

**Table 3:** Irrigation quotas and irrigation efficiency under SUI and FLI (CK1).

Year (a)	Irrigation method	Each irrigation amount (m^3^)	Irrigation times	Irrigation quota (m^3^·ha^-1^)	Yield of cherry (kg·ha^-1^)	Irrigation water productivity (kg·m^-3^·ha^-1^)
2009	SUI	320.0	2	640.0±38.5^a^	14057.3±788.5^a^	21.96±2.14^a^
	FLI(CK1)	720.0	2	1440.0±67.3^b^	13916.7±734.1^b^	9.66±0.89^b^
2010	SUI	320.0	3	960.0±43.9^a^	13156.2±694.2^a^	13.7±1.15^a^
	FLI(CK1)	720.0	3	2160.0±78.5^b^	12485.7±631.8^b^	5.78±0.57^b^

Different small letter means *P*<0.01. Changes in soil moisture content for different SUI and FLI quotas

The soil moisture contents for different SUI quotas are presented in Table 4. The soil moisture content for every layer increased with increases in irrigation quotas, which were significantly higher than those from CK2. The average values for soil moisture contents for SUI in 2009 were 15.8%-24.5% higher for the 0-20 cm layer, 17.1%-24.8% higher for the 21-50 cm layer, and 14.1%-24.0% higher for the 51-80 cm layer than those for CK2. The ANOVA indicated that there were no significant differences between them for the 81-110 cm soil layer (*P*>0.05), whereas the soil moisture contents for all treatments for the other soil layers were dramatically higher in significance (for 0-20 cm *P*<0.01; for 21-50 cm *P*<0.01; for 51-80 cm *P*<0.01) than those from CK2. These results indicated that water infiltration depths for the 80 m^3^ ha^-1^ treatment were 50 vertical cm, while infiltration depths for the other treatments were 80 cm. The average values of soil moisture content for SUI in 2010 were 19.3%-25.5% higher in the 0-20 cm layer, 18.8%-29.5% higher in the 21-50 cm layer, and 4.2%-14.3% higher in the 51-80 cm layer than those for CK2 (Table 4). The ANOVA indicated that there was no significant difference between treatments in the 81-110 cm soil layer (*P*>0.05), whereas the soil moisture content for all treatments in the 0-20 cm layer and the 21-50 cm were extremely significantly different (for 0-20 cm *P*<0.01; for 21-50 cm *P*<0.01) than those for CK2. In the 51-80 cm soil layer, the soil moisture content under the 80 m^3^ ha^-1^ treatment was not higher than that for CK2 (*P*>0.05), and the differences between the other treatments were significant (*P*<0.05).

**Table 4:** Soil moisture content for the different SUI and FLI quotas.

Irrigation method	Irrigation quotas (m^3^·ha^-1^)	Soil moisture content (%)							
		0-20(cm)		21-50(cm)		51-80(cm)		81-110(cm)	
		2009^†^	2010^‡^	2009	2010	2009	2010	2009	2010
SUI	80	17.56±1.54^b^	11.71±1.04^b^	18.75±1.72^c^	12.85±1.12^b^	14.22±1.34^b^	10.95±1.12^b^	11.51±1.25^a^	9.89±0.93^a^
	150	18.30±1.61^a^	11.96±1.15^b^	19.37±1.82^b^	13.52±1.21^a^	15.13±1.40^a^	11.57±1.30^ab^	11.62±1.30^a^	9.89±0.90^a^
	230	18.61±1.70^a^	12.18±1.27^a^	19.57±1.87^b^	13.75±1.32^a^	15.36±1.45^a^	11.86±1.38^a^	11.69±1.34^a^	9.90±0.98^a^
	320	18.89±1.78^a^	12.32±1.32^a^	20.98±1.89^a^	14.01±1.27^a^	15.45±1.49^a^	12.01±1.41^a^	11.87±1.39^a^	9.91±0.97^a^
FLI	80	15.68±1.29^d^	10.38±1.09^cd^	16.87±1.55^e^	11.26±1.09^cd^	12.97±1.31^cd^	10.73±1.17^cd^	11.22±1.21^a^	9.88±0.92^a^
	150	16.37±1.58^bc^	10.56±1.12^bc^	17.42±1.63^de^	11.93±1.22^c^	13.23±1.37^c^	11.28±1.28^bc^	11.51±1.24^a^	9.89±0.90^a^
	230	16.89±1.60^bc^	10.92±1.30^bc^	17.97±1.69^d^	12.18±1.28^b^	13.97±1.40^b^	11.65±1.35^b^	11.77±1.30^a^	9.91±0.97^a^
	320	17.27±1.63^b^	11.28±1.33^b^	18.39±1.77^cd^	12.96±1.30^b^	14.27±1.42^b^	11.97±1.43^b^	11.91±1.34^a^	9.91±0.97^a^
CK2	0	15.17±1.46^d^	9.82±0.78^d^	16.01±1.44^e^	10.82±0.96^d^	12.46±1.28^d^	10.51±0.93^d^	11.13±1.27^a^	9.88±0.96^a^

Different small letter means *P*<0.05; “†” The soil moisture content is the average values for the different SUI and FLI quotas on 26 April and 18 May in 2009; “‡ ” The soil moisture content is the average values for the different SUI and FLI quotas on 15 April, 20 May and 5 June in 2010. CK2 means the irrigation quotas is 0 m^3^·ha^- 1^ (no-water irrigation).

Table 4 showed that the average values of soil moisture content for FLI in 2009 were 3.4%-13.8% higher in the 0-20 cm layer, 5.4%-14.9% higher in the 21-50 cm layer, and 4.1%-12.1% higher in the 51-80 cm layer than those for CK2. The average values of soil moisture content for FLI in 2010 were 5.7%-14.9% higher in the 0-20 cm layer, 4.1%-19.8% higher in the 21-50 cm layer, and 2.1%-12.4% higher in the 51-80 cm layer than those for CK2 (Table 4). The ANOVA indicated that there was no significant difference between them for the 51-80 cm and for the 81-110 cm soil layers (for 51-80 cm *P*>0.05; for 81-110 cm *P*>0.05), whereas the soil moisture contents for all treatments for the other soil layers were significantly higher (for 0-20 cm *P*<0.01; for 21-50 cm *P*<0.01) than those for CK2.

Moreover, as shown in Table 4, the average values for soil moisture content from SUI in 2009 were 9.4%-12.0% higher for the 0-20 cm layer and 8.6%-11.1% higher for the 21-50 cm layer than those for the FLI treatment. In 2010, they were 9.2%-12.8% higher in the 0-20 cm layer and 8.1%-14.1% higher in the 21-50 cm layer than those for FLI. The ANOVA indicated that there were significant differences between the treatments in the 0-20 cm and the 21-50 cm soil layers (for 0-20 cm *P*<0.01; for 21-50 cm *P*<0.01), whereas the soil moisture contents for all of the treatments in the other soil layers were not significantly different (for 51-80 cm *P*>0.05; for 81-110 cm *P*>0.05).

### Growth and yield of cherries under different SUI and FLI quotas

The growth and yield of cherry trees under different SUI quotas were presented in Table 5. According to investigations at the end of the period of completed growth in 2009 and 2010, the mean diameter at breast height of cherry trees under SUI in 2009 was 14.1%-70.4% greater than that for CK2, and in 2010, it was 13.2%-75.0% greater than that for CK2. The mean growth of new branches for SUI in 2009 was 15.6%-89.0% higher than that for CK2, and in 2010, it was 11.4%-90.4% higher than that for CK2. The average cherry yield using SUI in 2009 was 10.4%-30.7% higher than for CK2, and in 2010, it was 8.7%-34.9% higher than for CK2. The ANOVA indicated that the differences between the diameter at breast height and the growth of new branches between all treatments and CK2 were significant (*P*<0.05). These results indicated that water supplemented with SUI could promote growth in cherry trees during a key period of water demand and that the positive effects were increased with increases in irrigation quotas.

**Table 5:** Cherry growth conditions and yields for the different SUI and FLI quotas in 2009 and 2010.

Year (a)	Irrigation method	Irrigation quotas (m^3^·ha^-1^)	Mean diameter at breast height (cm)	Mean growth new branch (cm)	Mean yield of cherry (kg·ha^-1^)
2009	SUI	80	0.81±0.10^cd^	19.91±1.32^d^	12782.6±656.1^d^
		150	0.91±0.13^b^	24.59±1.76^c^	13015.8±719.5^cd^
		230	1.15±0.15^a^	29.25±2.10^ab^	15141.5±814.9^a^
		320	1.21±0.18^a^	32.55±2.62^a^	14857.3±738.7^a^
	FLI	80	0.76±0.10^cd^	18.71±1.42^d^	11996.8±618.2^e^
		150	0.83±0.12^bc^	23.27±1.63^c^	12373.2±698.3^e^
		230	0.99±0.13^b^	28.38±2.35^b^	14337.1±807.7^b^
		320	1.10±0.16^ab^	30.71±2.97^a^	13246.9±717.9^c^
	CK2	0	0.71±0.08^d^	17.22±1.11^e^	11583.3±557.1^f^
2010	SUI	80	0.77±0.09^c^	17.62±1.19^e^	11010.7±510.4^e^
		150	0.90±0.07^b^	21.38±1.50^d^	12156.8±605.3^c^
		230	1.07±0.11^a^	27.32±2.44^b^	13414.4±756.5^a^
		320	1.19±0.16^a^	30.12±2.53^a^	13656.2±767.3^a^
	FLI	80	0.71±0.08^cd^	16.39±1.37^ef^	10374.2±525.1^f^
		150	0.87±0.06^b^	20.32±1.78^d^	11637.4±588.3^d^
		230	0.97±0.10^b^	25.17±2.68^c^	12198.7±724.1^c^
		320	1.08±0.14^a^	28.44±2.79^ab^	12893.5±758.7^b^
	CK2	0	0.68±0.07^d^	15.82±1.02^f^	10124.9±508.6^f^

Different small letter means *P*<0.05.

Cherry yields under all treatments were significantly higher than those in CK2 (Table 5). In 2009, the highest cherry yield was 15,141.5 kg ha^-1^, under the 230 m^3^ ha^-1^ treatment, it was 30.7% higher than yields for CK2, and the difference was significant (*P*<0.01). The cherry yield under the 320 m^3^ ha^-1^ treatment was 28.3% higher than that for CK2, and under the 150 m^3^ ha^-1^ treatment, it was 12.4% higher than that for CK2, and the differences were all extremely significant (*P*<0.01). Under the 80 m^3^ ha^-1^ treatment, the cherry yield was 10.4% higher than that for CK2, and the difference was significant (*P*<0.05). In 2010, the highest cherry yield was 13,656.2 kg ha^-1^, under the 320 m^3^ ha^-1^ treatment, 34.9% higher than that for CK2 (*P*<0.01). The cherry yield under the 230 m^3^ ha^-1^ treatment was 32.5% higher than that for CK2, and under the 150 m^3^ ha^-1^ treatment, it was 20.1% higher than that for CK2, and the differences were all extremely significant (*P*<0.01). Under the 80 m^3^ ha^-1^ treatment, the cherry yield was 8.7% higher than that in CK2, and the difference was not significant (*P*>0.05).

The growth and yield of cherry trees under different FLI quotas are presented in Table 5. The mean diameter at breast height of cherry trees under FLI in 2009 were 7.0%-54.9% greater than those for CK2, and in 2010, they were 4.4%-58.8% greater than those for CK2. The mean growth of new branches using FLI in 2009 was 8.7%-78.3% higher than that for CK2, and in 2010, it was 3.6%-79.8% higher than that for CK2. The average cherry yield for FLI in 2009 was 3.6%-23.8% higher than that for CK2, and in 2010, it was 7.4%-27.3% higher than that for CK2.

Moreover, the mean diameters at breast height for cherry trees under SUI in 2009 were 6.6%-16.2% greater than those for FLI, and in 2010, they were 8.5%-10.3% greater than those for FLI (Table 5). The mean growth of new branches of SUI in 2009 was 6.0%-11.8% higher than that for FLI, and in 2010, it was 5.2%-8.5% higher than that for FLI. The average cherry yield for SUI in 2009 was 5.2%-12.2% higher than that for FLI, and in 2010, it was 4.5%-10.0% higher than that for FLI.

## Discussion and Conclusions

### Effects of SUI and FLI on soil bulk density and soil porosity in cherry orchards

Soil porosity has a direct effect on soil aeration, permeability to irrigation water and the ability of fruit tree roots to penetrate the soil [25,26]. Moreover, it performs important regulatory functions for soil water, fertilizers, gases, heat and biological activity [27]. Compared with furrow irrigation, the soil bulk density and soil porosity of SUI in solar greenhouses were 21.2% lower and 29.0% higher, respectively [28,29]. In our study, the soil bulk density of SUI was significantly lower than that of FLI, and the total soil porosity, capillary porosity and non-capillary porosity of SUI were all significantly higher than those of FLI, respectively (Table 2). In particular, the differences in the 0-20 cm soil layer were most significant. The reason might be that SUI is capable of applying small amounts of water directly to the plant root zone, where water is needed. At the same time, the destructive effects of SUI on soil structure are minor, and this allows for surface soils to be maintained in a loose condition. However, under FLI, the soil water status becomes saturated or supersaturated in a short time, and therefore, large amounts of gravitational water are produced, and the soil’s aggregate structure is easily destroyed when immersed in large amounts of irrigation water. These observations indicated that SUI had a greater ability to maintain soil structure than did FLI.

### Effects of SUI and FLI on soil temperature in cherry orchards

Compared with furrow irrigation, soil temperatures under SUI in solar greenhouses were 1.4-3.0°C higher [30,31]. In our present study, the soil temperatures under SUI at different soil depths were higher than those for FLI, and the maximum value was 4.1°C and occurred on the soil surface at 13: 00 (Figure 3). Moreover, these results indicated that the differences in soil temperature at 0 cm and 5 cm of soil depth for SUI and FLI were extremely significant (*P*<0.01) and that significant differences existed at the 10 cm soil depth (*P*<0.05), whereas no significant differences were observed at 15 cm and 20 cm soil depths (*P*>0.05). The reason for these results might be that the soil under FLI becomes saturated with water in a short time, leading to a lower soil temperature than under SUI.

### Effects of SUI and FLI on soil moisture content and irrigation productivity in cherry orchards

At present, studies on soil water movement and water use efficiency using SUI for vegetable production in solar greenhouses or in the field have been reported [27,28,32]. However, few studies have focused on soil water movement and water use efficiency when using SUI in orchards. In our present study, the soil moisture content of different soil layers when using SUI increased with increases in irrigation quotas. Moreover, the results showed that the average values for soil moisture content under SUI were significantly higher in the 0-20 cm layer and in the 21-50 cm layer than were those under FLI (for 0-20 cm *P*<0.01; for 21-50 cm *P*<0.01), whereas the soil moisture contents for all treatments in other soil layers were not significantly different (for 51-80 cm *P*>0.05; for 81-110 cm *P*>0.05) (Table 4 and Table 5).

When compared with FLI, the average water-saving efficiency of SUI was 55.6%, and the irrigation productivity of SUI increased by 7.9-12.3 kg m^3^ ha^-1^ (Table 3). These results indicated that the water-saving effects and water use efficiency of SUI were higher than those for FLI. Under SUI, irrigation water infiltrated slowly into the soil slowly via pipes, a portion of the water moved upward by capillary permeability, and a large portion of the water moved down into the subsoil due to gravity. The soil moisture content levels at different depths for SUI were between the wilting moisture content level and the field capacity (Table 1 and Table 4). Seventy-five percent of the irrigation water was distributed in the 10-50 cm soil layer, 10% was in the 0-10 cm soil layer, and 15% was in the 50-80 cm soil layer. No irrigation water infiltrated below 80 cm of depth; therefore, there was no deep percolation and there was low evaporation from the soil, and soil moisture was primarily distributed in the 10-50 cm soil layer under SUI. SUI could therefore enhance the absorption and utilization of soil moisture by the roots of fruit trees and improve the utilization and production efficiency of irrigation water.

### Effects of SUI and FLI on growth conditions and yield of cherries

SUI can maintain the physical structure of soil and improve soil temperature and moisture contents in the root zone, promoting the growth and development of cherry trees. In our present study, water supplemented via SUI during key water demand periods had obvious effects. The mean growth of new branches when using SUI was 11.4%-90.4% higher than for CK2, and the average cherry yield for SUI was 8.7%-34.9% higher than that for CK2 in both 2009 (normal rainfall year) and 2010 (low rainfall year) (Table 5). Moreover, compared with FLI, the mean growth of new branches under SUI was 5.2%-11.8% higher than under FLI, and the average cherry yields for SUI were 4.5%-12.2% higher than those for FLI. In 2009, the highest cherry yields (15,141.5 kg ha^-1^) were under the 230 m^3^ ha^-1^ treatment, which were 30.72% higher than those for CK2. The cherry yield under the 320 m^3^ ha^-1^ treatment was lower than that for 230 m^3^ ha^-1^. The reason might be that excessive quantities of irrigation water can result in overgrowth, fruit abscission and low fruit yield.

Therefore, it is appropriate to irrigate 2-3 times, with 230 m^3^ ha^-1^ per irrigation, using SUI, during a year of normal rainfall, and irrigation times and irrigation quotas should increase proportionately during a low rainfall year. There is no significant surface evaporation and deep percolation under SUI; therefore, SUI can better maintain a soil’s physical properties and structure, greatly improve irrigation water use efficiency, and significantly increase fruit yield in hilly semi-arid areas of northern China.
